# A novel compound mutation in *GLRA1* cause hyperekplexia in a Chinese boy- a case report and review of the literature

**DOI:** 10.1186/s12881-017-0476-6

**Published:** 2017-10-06

**Authors:** Zhiliang Yang, Guilian Sun, Fang Yao, Dongying Tao, Binlu Zhu

**Affiliations:** Department of Pediatrics, The First Hospital of China Medical University, No. 155 Nanjing North Street, Heping District, Shenyang, 110001 Liaoning Province People’s Republic of China

**Keywords:** Hyperekplexia, Startle disease, *GLRA1*, Phenotype

## Abstract

**Background:**

The pathogenesis of hereditary hyperekplexia is thought to involve abnormalities in the glycinergic neurotransmission system, the most of mutations reported in *GLRA1*. This gene encodes the glycine receptor α1 subunit, which has an extracellular domain (ECD) and a transmembrane domain (TMD) with 4 α-helices (TM1–TM4).

**Case presentation:**

We investigated the genetic cause of hyperekplexia in a Chinese family with one affected member. Whole-exome sequencing of the 5 candidate genes was performed on the proband patient, and direct sequencing was performed to validate and confirm the detected mutation in other family members. We also review and analyse all reported *GLRA1* mutations. The proband had a compound heterozygous *GLRA1* mutation that comprised 2 novel *GLRA1* missense mutations, C.569C > T (p.T190 M) from the mother and C.1270G > A (p.D424N) from the father. SIFT, Polyphen-2 and MutationTaster analysis identified the mutations as disease-causing, but the parents had no signs of hyperekplexia. The p.T190 M mutation is located in the ECD, while p.D424N is located in TM4.

**Conclusions:**

Our findings contribute to a growing list *GLRA1* mutations associated with hyperekplexia and provide new insights into correlations between phenotype and *GLRA1* mutations. Some recessive mutations can induce hyperekplexia in combination with other recessive *GLRA1* mutations. Mutations in the ECD, TM1, TM1-TM2 loop, TM3, TM3-TM4 loop and TM4 are more often recessive and part of a compound mutation, while those in TM2 and the TM2-TM3 loop are more likely to be dominant hereditary mutations.

**Electronic supplementary material:**

The online version of this article (10.1186/s12881-017-0476-6) contains supplementary material, which is available to authorized users.

## Background

Hyperekplexia, also known as startle disease, is a rare disorder that is classically characterized by exaggerated startle responses to unexpected stimuli. It was first reported in 1958 [1]. This disorder can cause serious injuries due to frequent falls and may cause infantile death via induced apnoea. Hyperekplexia can be hereditary or can occur sporadically. It shows genetic heterogeneity, with the first causal mutations reported in *GLRA1* (glycine receptor (GlyR) alpha 1) in 1993 [2]. Mutations in 4 other genes have since been reported: *GLRB* (GlyR beta) [3–8], *GPHN* (gephyrin) [9], *ARHGEF9* (Cdc42 guanine nucleotide exchange factor 9) [10] and *SLC6A5* (solute carrier family 6 member 5) [11–14]. All of these genes encode proteins that are associated with the glycine transmission system. Recently, a clear correlation of mutation in β-catenin gene (*CTNNB1*) with an atypical syndromic hyperekplexia had been reported in a case of CTNNB1-related syndrome and *CTNNB1* was considered to be a cause gene for syndromic hyperekplexia [15].

The most of mutations have been reported in *GLRA1* [16, 17], which encodes the GlyR α1 subunit. This subunit contains an extracellular domain (ECD) and a transmembrane domain (TMD) that comprises 4 α-helices, termed TM1–TM4. The mutation loci have been reported to be associated with recessive or dominant heredity [17]. Here we report a case of genetically confirmed hyperekplexia caused by two novel *GLRA1* mutations, which together constitute a compound mutation, that were inherited from the proband’s unaffected parents. We also discuss possible correlations between mutation loci and hyperekplexia phenotype.

## Case presentation

**Fig. 1:**
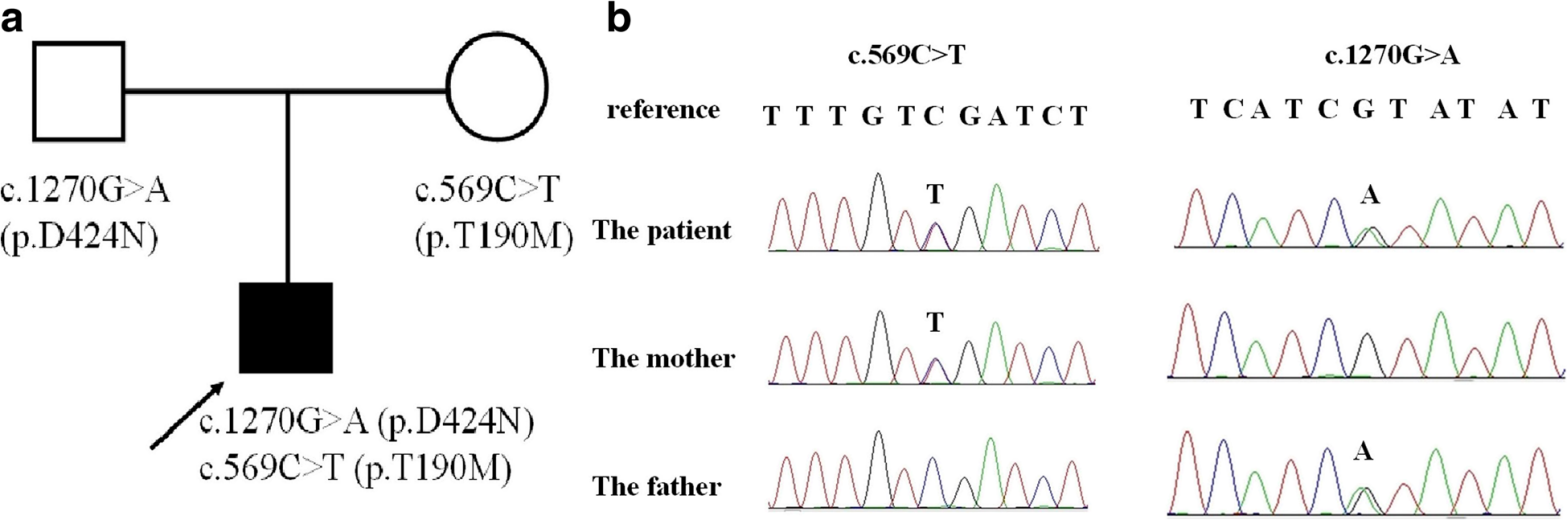
The family pedigree showing the mutations detected in *GLRA1*. **a** The pedigree of the family with hyperekplexia. The arrow indicates the proband; his parents have no signs of hyperekplexia. **b** The mutations detected in the family. The proband has both mutations, while the c.569C > T mutation was only detected in his mother and the c.1270G > A mutation was only detected in his father

The proband (Fig. 1a) was a 13-year-old Chinese boy who was admitted to hospital with a chief complaint of frequent falls in response to sudden stimuli for about 13 years. He was born at term and had an unremarkable antenatal and birth history. In the neonatal period, he showed body rigidity and trembling for a few seconds in response to vocal stimulation but did not show apnoea, and his parents paid little attention at the time. He showed no developmental delays or neurologic deficits, but seemed “timid” when he began to walk at age 1 year and showed body stiffening in response to sudden audible, visual or tactile stimulation, sometimes falling down and becoming pale but without losing consciousness. He often had superficial facial injuries in kindergarten and in primary school. When he was 10 years old, he fell and broke his right forearm, and twice he fell down stairs in response to sudden noises. After that, he was accompanied by a family member when he went outside or walked up and down stairs. About 1 month prior to admission, he fell after being startled and broke his left forearm.

The boy was admitted to several other hospitals over the years. His brain MRI and MRA, 24-h electroencephalogram (EEG), electrocardiogram (ECG) and transcranial Doppler ultrasound apparatus (TCD) results were normal. He was diagnosed with possible epilepsy and prescribed antiepileptics; however, he showed no improvement after 3 months, so the antiepileptics were withdrawn. Physical examination showed a cautious gait with a wide stride, and the nose-tapping test (head-retraction reflex) was positive. The IQ (intelligence quotient) using the Wechsler Intelligence Scale for Children was 89. To exclude epilepsy, a video EEG was performed. During the test, some stimuli were presented, but no abnormal waves were induced. His ceruloplasmin and lactic acid levels were normal. Hyperekplexia was considered based on his medical history and on repeated normal testing results from several hospitals.

After genetic counselling with a clinical geneticist, genetic analysis for hyperekplexia was performed after obtaining the approval of the ethics committee of the First Hospital of China Medical University. With written consent from his parents, peripheral blood samples were collected from the proband and his parents. DNA was extracted using the Puregene Extraction Kit (Qiagen, Germany).The Agilent SureSelect Human Exon Capture Sequencing platform was used to screen DNA from the proband for the 5 genes involved in the glycinergic neurotransmission system on DNA i.e. *GLRA1*, *GLRB*, *GPHN*, *ARHGEF9* and *SLC6A5*.The obtained data were analysed using Agilent SureSelect Human All Exon V5 software, and the variants were called according to the protocol for the platform. The variants were interpreted according to the guidelines from American College of Medical Genetics and Genomics and patient phenotype [18]. The detected missense mutations were validated using direct sequencing. Direct sequencing was performed on DNA from individuals of all family members using the 3730xl DNA Analyzer (Applied Biosystems, Foster City, CA, USA), and the samples were subjected to sequence analysis using Sequence Scanner v1.0 (Applied Biosystems, Foster City, CA, USA). The sequencing procedure or mutation validation were performed by Hicetech Test Laboratory (Beijing, China) which provides the third party inspection services.

The hypothetical effects of the mutations on protein function were analysed using the Polymorphism Phenotyping v2 (PolyPhen-2) prediction tool (http://genetics.bwh.harvard.edu/pph2/dbsearch.shtml), SIFT (http://sift.jcvi.org/www/SIFT_enst_submit.html) and MutationTaster (http://www.mutationtaster.org/index.html).

Almost all hyperekplexia cases respond well to clonazepam (CZP). CZP is a γ-aminobutyric acid receptor alpha1(GABARA1) agonist and can enhance GABA-gated chloride channel function. The glycine receptor and GABA receptor are members of same superfamily of ligand-gated ion channels and share common transmembrane topology, structural and functional features. CZP was presumed to compensate for the defective glycine-gated chloride channel function by enhancing GABA-gated chloride channel function in hyperekplexia [19].

In our patient, after the *GLRA1* mutations were identified and hyperekplexia was diagnosed, CZP administration was initiated at 0.02 mg/kg per day, administered in 3 doses, with slight increases every 3 day. After 1 week, the daily dose was 0.05 mg/kg, and his parents reported that the startle response had almost disappeared, but the patient appeared sleepy all day. The dose of CZP was unchanged, and at the 6-month follow-up, the startle responses were almost resolved.

The proband patient had 2 heterozygous missense point mutations in *GLRA1* (C.569C > T (p.T190 M) and C.1270G > A (p.D424N) (reference sequence: NM_001146040) that were validated with direct sequencing. His mother had the C.569C > T (p.T190 M) mutation, and his father had the C.1270G > A (p.D424N) mutation (Fig. 1b). The p.T190 M mutation is located in the ECD, while p.D424N is located in TM4. PolyPhen-2, SIFT and MutationTaster analysis suggested that both of these mutations would negatively affect gene function (Table 1).

**Table 1:** Functional evaluation of the *GLRA1* mutations detected in the family of a Chinese boy with hyperekplexia

Base change	Exon number	Amino acid change	PolyPhen-2 analysis	SIFT analysis	MutationTaster analysis
c.569C > T	6	p.T190M	Probably damaging	Damaging	Disease causing
c.1270G > A	9	p.D424N	Probably damaging	Damaging	Disease causing

**Fig. 2:**
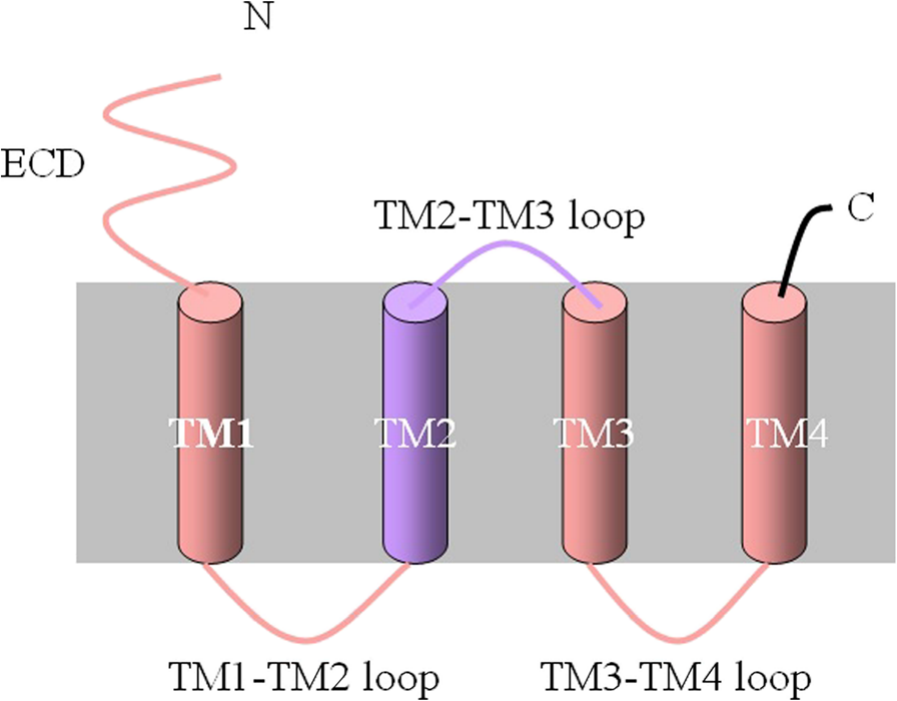
The schematic diagram shows the spanning domains (TM1-TM4) topology of GlyR α1 subunit. Mutations in the ECD, TM1, TM1-TM2 loop, TM3, TM3-TM4 loop and TM4 are more often recessive and part of a compound mutation, while those in TM2 and the TM2-TM3 loop are more likely to be dominant hereditary mutations

We next reviewed reported *GLRA1* mutations in hyperekplexia according to mutation location (Table 2). About 67.7% (42/62) are recessive, about 29% (18/62) are dominant and about 3.2% (2/62) are novel mutations. Among the recessive mutations, about 52.4% (22/42) are compound mutations (i.e. present in combination with another mutation). The compound mutation in our patient was first reported, we cannot define whether it is dominant or recessive. The mutations were mostly located in the ECD (21/62) and TM2 (13/62), with only 1 mutation in the TM1-TM2 loop and 2 in TM3. About 90.4% (19/21) of the mutations in the ECD domain, about 30.8% in the TM2 domain, all in TM3 and the TM3-TM4 loop, and 75% in TM4 were recessive. The only mutation in the TM1-TM2 loop was dominant; while 69.2% of the mutations in the TM2 domain and about 66.7% in the TM2-TM3 loop were dominant (Fig. 2). The mutations and referenced studies are shown in Additional file 1: Table S1.

**Table 2:** Distribution of hyperekplexia mutations in GLRA1 according to the hGlyR position

hGlyR position	Mutations *n*	Recessive mutations *n* (%)	Recessive mutations that are compound mutations *n* (%)	Reported dominant mutations *n* (%)	Reported de novo mutations *n* (%)
NA	2	2 (100)	1 (50)	0	0
ECD	21	19 (90.4)	10 (52.6)	1 (4.8)	1 (4.8)
TM1	7	5 (71.4)	2 (40)	2 (28.6)	0
TM1-TM2 loop	1	0	–	1 (100)	0
TM2	13	4 (30.8)	1 (25)	9 (69.2)	0
TM2-TM3 loop	6	1 (16.7)	0	4 (66.7)	1 (16.7)
TM3	2	2 (100)	2 (100)	0	0
TM3-TM4 loop	6	6 (100)	4 (66.7)	0	0
TM4	4	3 (75)	2 (66.7)	1 (25)	0
Total	62	42 (67.7)	22 (52.4)	18 (29)	2 (3.2)

*NA* not applicable, *ECD* extracellular binding domain, *TM* transmembrane domain, *n* number of mutations, *%* percentage of mutations

## Discussion and conclusions

Mutations in 5 candidate genes relating to the glycinergic neurotransmission system have been identified in hyperekplexia [2–14]). The *GLRA1* gene encodes the GlyR α1 subunit [2], *GPHN* encodes gephyrin [9], *ARHGEF9* encodes collybistin [10] and *SLC6A5* encodes the glycine transporter GlyT2 [11]. Gephyrin and collybistin are involved in GlyR clustering, while the glycine transporter is a presynaptic NaCl-dependent transporter. Some gene-negative cases in the 5 genes have been reported, raising the possibility that there are other candidate genes [20], and *CTNNB1* was reported recently [15], the event indicate hyperekplexia could involve multiple genes.

We reviewed all of the *GLRA1* mutations in hyperekplexia that have been reported to date and found that 29% are dominant missense and 67.7% are recessive missense mutations. This is in accordance with a report that 23% of the mutations are dominant missense, 39% are recessive missense and 38% are recessive nonsense [20]. Our review found that mutations more often occurred in the ECD and TM2 domains; that mutations in the ECD, TM1, TM1-TM2 loop, TM3, TM3-TM4 loop and TM4 were more likely to be recessive and compound mutations (with other heterozygous mutations); and that few mutations were located in TM2 and the TM2-TM3 loop, but these were more likely to be dominant pathogenic mutations.

Our patient had two heterozygous mutations, C.569C > T(p.T190 M) from his mother, located in the ECD, and C.1270G > A(p.D424N) from his father, located in TM4. Neither were located in regions of the gene in which they were likely to be dominant hereditary mutations. Indeed, neither parent had hyperekplexia, even in silico analysis predicted that each mutation would be disease causing. However, the proband patient, who had both mutations, had hyperekplexia. This suggests that these recessive mutations of the GLRA1 gene in a compound heterozygote state are pathogenic and cause hyperekplexia.

The missense heterozygous mutations in the parents of the proband were autosomal recessive, even though the analysis found that they could cause abnormal protein function. It is possible that having a normal allele produces sufficient protein to allow normal glycinergic neurotransmission. When the patient inherited both mutations from his parents, the deleterious effects of the mutated proteins could not be counteracted by an unaffected allele.

Some mutant proteins are recognized by the endoplasmic reticulum (ER) control system and broken down via proteasomes [21]. The mutated proteins were not released into the blood and were not active, which could explain why the mutations are recessive hereditary mutations. If the mutated proteins bypass the ER control system, they are transported into the blood and trigger an autoimmune response. Notably, GAD65 (glutamic acid decarboxylase 65-kilodalton isoform, glutamate decarboxylase 2) and GlyR autoimmunity may result in stiff-person syndrome or in progressive encephalomyelitis with rigidity and myoclonus (PERM) [22]. A striking feature of PERM with GlyR autoantibodies is a pathologically exaggerated startle response that resembles hyperekplexia [23]. We did not test our patient for relevant autoantibodies as we did not have access to the technology.

In conclusion, hyperekplexia is potentially treatable, and should be treated in order to prevent injuries and improve the quality of life. Early diagnosis and treatment is important. The location of the *GLRA1* mutation correlates hyperekplexia, and gene-negative cases of hyperekplexia suggest that other genes may be involved in this disorder. Although hyperekplexia is a clinical diagnosis, we suggest that prompt genetic analysis may be useful for early definite diagnosis of hyperekplexia and subsequent preconception counselling and safer care for affected neonates.

## Additional file

Additional file 1: Table S1. List of hyperekplexia mutations in GLRA1 according to the hGlyR position. References of individual studies for mutations in GLRA1 gene are summarized in the Supplementary Table. NA, not applicable; ECD extracellular binding domain; TM, transmembrane domain; n, number of mutations. (DOC 306 kb)

## Abbreviations

CZP: Clonazepam
ECD: Extracellular domain
GABA: γ-Aminobutyric acid
GlyR: Glycine receptor
TMD: Transmembrane domain
