# Supplementary material for: A novel compound mutation in GLRA1 cause hyperekplexia in a Chinese boy- a case report and review of the literature
Source: BMC Med Genet. 2017 Oct 6;18:110. doi: 10.1186/s12881-017-0476-6 (PMC5631533; doi:10.1186/s12881-017-0476-6)
Supplement: Additional file 1: Table S1. — List of hyperekplexia mutations in GLRA1 according to the hGlyR position. References of individual studies for mutations in GLRA1 gene are summarized in the Supplementary Table. NA, not applicable; ECD extracellular binding domain; TM, transmembrane domain; n, number of mutations. (DOC 306 kb) [file 12881_2017_476_MOESM1_ESM.doc]

Additional file 1: Table S1. List of hyperekplexia mutations in GLRA1 according to the hGlyR position.
Studies
(years)	hGlyR position	Inheritance	Mutation type	Mutation 	Compound mutation	Position of compound mutation	
Brune et al.
(1996)	NA
(n=2)	Recessive
(n=2)	Deletion	ÄEx1–7	-	-	
Chung et al.
(2010)				ÄEx4–7	Heterozygous R65L	ECD	
Chung et al.
(2010)	ECD
(n=21)	Recessive
(n=19)	Missense	R65L	ÄEx4–7	NA	
Chung et al.
(2010)				R65W	Heterozygous P230S	TM1	
Tsai et al.
(2004)				W68C	Heterozygous R316X	TM3-TM4 loop	
Chung et al.
(2010)				E103K	Heterozygous L184fsX21	ECD	
Chan et al.
(2012)				C138S	Heterozygous D148fsX16	ECD	
Present case				T190M	Heterozygous D424N	TM4	
Chung et al.
(2010)			Nonsense	Y197X	Heterozygous Y202X	ECD	
Rees et al.
(2001)				Y202X	Heterozygous Y197X	ECD	
Horváth et al.
(2014)			Missense	I43F	-	-	
Coto et al.
(2005)				R72H	-	-	
Bode et al.
(2013)				R72C	-	-	
Rees et al.
(2001)				M147V	-	-	
Chung et al.
(2010)				D165G	-	-	
Al-Futaisi et al.
(2012)				W170S	-	-	
Bode et al.
(2013)				R218W	-	-	
Rees et al.
(2001)			Deletion	R72fsX47	-	-	
Zoons et al.
(2012)				K132fsX15	-	-	
Table S1. List of hyperekplexia mutations in GLRA1 according to the hGlyR position (continued).
Reference	hGlyR position	Inheritance	Mutation type	Mutation 	Compound mutation	Position of compound mutation	
Chan et al.
(2012)				D148fsX16	Heterozygous C138S	ECD	
Chung et al.
(2010)				L184fsX21	Heterozygous E103K	ECD	
Chung et al.
(2010)		Dominant 
(n=1)	Missense	Y128C	-	-	
del Giudice et al.
(2001)		De novo
(n=1)	Missense	R218Q	Heterozygous S296X	TM3	
Bode et al.
(2013)	TM1
(n=7)	Recessive
(n=5)	Missense	P230S	Heterozygous R65W	ECD	
Chung et al.
(2010)				S231N	Heterozygous S296X	TM3	
Forsyth  et al.
(2007)				Y228C	-	-	
Humeny   et al.
(2002)				S231R	-	-	
Rees et al.
(1994)				I244N	-	-	
Bode et al.
(2013)		Dominant
(n=2)		Q226E	-	-	
Gilbert et al.
(2004)				W239C	-	-	
Saul et al.
(1999)	TM1-TM2 loop (n=1)	Dominant
(n=1)	Missense	P250T	-	-	
Vergouwe  et al.
(1999)	TM2
(n=13)	Recessive
(n=4)	Missense	R252H	Heterozygous R392H	TM4	
Chung et al.
(2010)				R25C	-	-	
Chung et al.
(2010)				G254D	-	-	
Lapunzina et al.
(2003)				S270T	-	-	

Table S1. List of hyperekplexia mutations in GLRA1 according to the hGlyR position (continued).
Reference	hGlyR position	Inheritance	Mutation type	Mutation 	Compound mutation	Position of compound mutation	
del Giudice et al.
(2001)		Dominant
(n=9)		V260M	-	-	
Chung et al.
(2010)				T265I	-	-	
Milani  et al.
(1996)				Q266H	-	-	
Becker et al.
(2008)				S267N	-	-	
Shiang et al.
(1993)				R271L	-	-	
Shiang et al.
(1993), Mine  et al. (2015)				R271Q	-	-	
Gregory   et al. (2008)				R271P	-	-	
Mine  et al. (2015)				A272P	-	-	
Lee et al.
 (2013)			Nonsense	R271X	-	-	
Gilbert et al.
(2004)	TM2-TM3
Loop (n=6)	Recessive
(n=1)	Nonsense	Y279X	-	-	
Elmslie et al.
(1996)		Dominant
(n=4)	Missense	K276E	-	-	
Shiang et al.
(1995)				Y279C	-	-	
Poon et al.
(2006)				Y279S	-	-	
Bode et al.
(2013)				V280M	-	-	
Kang et al.
(2008)		De novo
(n=1)	Missense	K276Q	-	-	


Table S1. List of hyperekplexia mutations in GLRA1 according to the hGlyR position (continued).
Reference	hGlyR position	Inheritance	Mutation type	Mutation 	Compound mutation	Position of compound mutation	
Bode et al.
(2013)	TM3
(n=2)	Recessive
(n=2)	Missense	L291P	Heterozygous D388A	TM3-TM4 loop	
Bellini et al.
(2007)			Nonsense	S296X	Heterozygous S231N
           R218Q	TM1
ECD	
Mine  et al. (2015)	TM3-TM4 loop(n=6)	Recessive
(n=6)	Missense	A384P	Heterozygous R392H	TM3	
Bode et al.
(2013)				D388A	Heterozygous L291P	TM3	
Jungbluth et al.
(2000)				G342S	-	-	
Tsai et al.
(2004)			Nonsense	R316X	Heterozygous W68C	ECD	
Mine  et al. (2015)				R316X	Heterozygous R392H	TM4	
Bode et al.
(2013)				E375X	-	-	
Vergouwe  et al.
(1999)	TM4
(n=4)	Recessive
(n=3)	Missense	R392H	Heterozygous R252H	TM2	
Hmami et al.
(2014)				R392H	-	-	
Present case				D424N	Heterozygous T190M	ECD	
Bode et al.
(2013)		Dominant
(n=1)		R414H	-	-	
NA, not applicable; ECD extracellular binding domain; TM, transmembrane domain; n, number of mutations.
